# 

**Published:** 1906-06-02

**Authors:** 


					TtLK'H USB1 TAIj.
T'** <C
OSRLTALr
J H0SBX1AJL
;cotY V/esterm Infirua'm    - - ?
NO. 1,027. Vol. XL. [afewTpfpen] SATURDAY,
June 2nd, 1906.
[ see'p!'i66Terma] price THREEPENCE

				

